# A systemic review and meta-analysis on the prevalence and associated factors of hypertension among adult clients in Ethiopia

**DOI:** 10.4314/ahs.v23i4.32

**Published:** 2023-12

**Authors:** Tadele Lankrew Ayalew, Belete Gelaw Wale, Bitew Tefera Zewudie

**Affiliations:** 1 Department of Nursing, school of nursing, college of health science and medicine Wolaita Sodo University, Sodo, Ethiopia; 2 Department Nursing, College of health sciences and medicine, Wolkite University, wolkite, Ethiopia

**Keywords:** Hypertension, adults, systemic review, meta-analysis, Ethiopia

## Abstract

**Background:**

Hypertension is a major risk factor for premature mortality and excessive morbidity in the world. It is a growing public health problem in developing countries including Ethiopia. It is a silent killer. Information on the prevalence of hypertension and its associated factors is to be considered vital to focus on early diagnosis and improve prevention and control of cardiovascular diseases. However, on the prevalence and contributing factors of hypertension in Ethiopia, there is a dearth of information. Thus, this review aimed to estimate the pooled prevalence of hypertension and its contributing factors among Ethiopia's adult population.

**Objective:**

The main objective of this study was to provide pooled evidence on the prevalence of hypertension among the adult population in Ethiopia.

**Methods and material:**

This systematic review and meta-analysis were searched through MEDLINE/ Pub Med, Cochrane Library, and Google Scholar by using different search terms on the prevalence of hypertension and Ethiopia. Joanna Briggs Institute Meta-Analysis of Statistics Assessment and Review Instrument was used for critical appraisal of studies. The analysis was done using STATA 14 software. The Cochran Q test and I^2^ test statistics were used to test the heterogeneity of studies. Egger's test was used to show the publication bias. The pooled prevalence of HDP and the odds ratio (OR) with 95% confidence interval were presented using forest plots.

**Results:**

A total of 22 studies with 14,202 participants were included in this review and the overall estimated prevalence of hypertension among the adult population in Ethiopia was 28.02% (95% CI (23.89%, 32.15%). Age 4.37(2.71, 6.04), sex (AOR=2.54, 95% CI: 1.00–4.09), family history of hypertension (AOR=3.05, 95% CI, 1.89, 4.21), inactive physical exercise (AOR=2.67, 95% CI: 1.38, 3.97), being obese (AOR=3.94, 95CI:2.83, 5.06), khat chewing (AOR=3.73, 95% CI: 2.65, 4.80), salt consumption (AOR=4.20, 95% CI: 1.55, 6.86) were significantly associated factors. Subgroup meta-analysis done by region showed that a higher in Tigray region 52.19(46.54, 57.66), and the lower was observed in Harare region1 2.71 (9.54, 15.87).

**Conclusion:**

The prevalence of hypertension among the adult population in Ethiopia is high. Healthcare professionals and other stakeholders should give attention to the early detection of hypertension in Ethiopia to reduce the burden of the disorder.

## Introduction

Hypertension is one of the main public health challenges of the disease globally. Hypertension is a state of elevated systemic blood pressure. Hypertension is a major risk factor for mortality and excessive morbidity throughout the world. It is a predisposing factor in the development of coronary disease (CAD), cerebrovascular disease (CEVD), Systemic atherosclerosis, congestive heart failure (CHF), chronic kidney disease, and dementia 1–3. Hypertension is a silent killer that most patients are detected incidentally when they are admitted to the hospital for unrelated diseases or preoperative medical check-ups 4–7.

Hypertension is diagnosed if, when it is measured on the different days, the systolic blood pressure reading on both days is > 140 mmHg and/ or a diastolic blood pressure reading on both days is > 90mmHg. In the globe, there is an estimate of 1.39 billion people have hypertension 8. Based on the recent publication on the update of pediatric clinical practice guideline by the American Academy of Pediatrics, the definition of hypertension in adolescents of age more than 13 years of age is a systolic > 130 mmHg and /or a diastolic of > 80mmHg 9,10. The prevalence of hypertension among adults from these were 31.1%, which was higher in low income (31.5%, 1.04 billion people) than in high-income countries (28.5%, 349 million) in the world 11.

The exact causes of hypertension are not known, but there are a lot of risk factors and conditions, which may play a great role in the development of hypertension around the globe. There are various factors such as an individual's age, smoking behavior, consuming alcohol for a long duration reported as associated factors with hypertension 12,13. The prevalence of hypertension among adult people is rising globally due to increasing different level risk factors, exposure of hypertension including unhealthy diet (high salt and low fruit and vegetable intake), physical inactivity, tobacco and alcohol consumption khat chewing, and obesity, and not having health checkups regularly 12,14,15. Still, the trend of the global prevalence of hypertension is dramatically increasing. Globally, at least one billion people have hypertension, and a projected figure of more than 1.5 billion expected by 2025^13^.

Well managed and well controlled hypertension leads to better quality of life and reduces the risk of complications, which include coronary artery disease, heart failure, and cerebrovascular disease, and chronic kidney disease. Early detection, early diagnosis, and maintaining a healthy lifestyle including promoting physical activities, avoiding smoking, and accurate medication have been shown to help prevent and control hypertension among adolescents 9,10,13.

The World Health Organization (WHO) predicted deaths from non-communicable diseases increases globally by 17% over the next ten years where the greatest increase will be in the African region (by 27% or 28 million deaths from NCDs. In Ethiopia, non-communicable diseases such as hypertension have begun to emerge as the leading causes of hospital admissions, mortality, and morbidity in health facilities located around the nation. Together, these sequels of hypertension exert a critical impact on public health, medical expense, and the health care system in the country.

Little is known about the magnitude and factors of hypertension in Ethiopia; an up-to-date and comprehensive assessment of the evidence concerning hypertension in Ethiopia is lacking. The previous systematic review and meta-analysis are neglected the prevalence of hypertension among pregnancy 16. Accordingly, both the previous systematic review and meta-analysis above you mentioned are focused only by depending on age category (all aged grouped >18). But this review focused on the prevalence of hypertension including both men and women who are diagnosed with hypertension and / or hypertensive disorders and associated factors among adult clients in Ethiopia, i.e., Studies conducted on pregnancy-induced hypertension, and hypertension prevalence reports on other comorbidities. Additionally, to increase the validity of this manuscript, all thesis papers, editorials, reviews, case-control, cohort, and randomized trial studies were included in this review by JBI screening criteria. Therefore, these points (including pregnancy and different study design) were the gap we identified from the previous review. On the other hand, urbanization is expanding, lifestyles are changing, the literacy rate is low, and people are still living in poverty. However, on the prevalence and contributing factors of hypertension in Ethiopia, there is a dearth of information. Thus, this review aimed to estimate the pooled prevalence of hypertension and its contributing factors among Ethiopia's adult population.

## Methods

### Study design and search strategy

This systematic review and meta-analysis focused on the prevalence of hypertension including both men and women including pregnancy women who are diagnosed with hypertension. On the other hand, to increase the validity of this manuscript, all thesis papers, editorials, reviews, case-control, cohort, and randomized trial studies were included in this review by JBI screening criteria on the prevalence of hypertensive disorders and associated factors in Ethiopia. The studies were retrieved through internet search from the databases of MEDLINE/Pub Med, Cochrane Library, and Google Scholar. This systematic review was done in line with the Preferred Reporting Items for Systematic and Meta-analysis (PRISMA) protocol 17 to estimate the prevalence of hypertensive and associated factors in Ethiopia. We checked the database (http://www.library.UCSF.edu) and the Cochrane library to ensure this had not been done before and to avoid duplication. We also checked whether there was any similar ongoing systematic review and meta-analysis in the PROSPERO database. PROSPERO Registration message on Sep 20, 2021, 9:22 PM; CRD [279853]; reassured that there had been no previous similar studies undertaken.

The search was performed by using Medical Subject Heading by considering the following search terms; prevalence, hypertensive disorders, associated factors, and Ethiopia. The reference lists of already identified studies were screened to retrieve articles. All included studies defined hypertension as Systolic Blood Pressure (SBP) >140 mmHg and /or a Diastolic Blood Pressure (DBP) >90 mmHg or known hypertensive patients on treatment. The search MeSH headings were hypertension and synonyms for hypertension were used. The synonyms of hypertension are “blood pressure high”, “and blood pressures, high”, “high blood pressure”, and “high blood pressures”. All articles up to 24 September 2021 were included in this review.

### Eligibility criteria

To declare the inclusion and exclusion criteria, we used CoCoPop (Condition, Context, and population) for prevalence studies.

Inclusion criteria of this review were as follows;

1). Included participants both men and women adult clients who are living in Ethiopia.

2). Study design was cross-sectional, cohort, case control, randomize trail etc.

3). All studies were conducted on hypertensive disorders and associated factors among adult clients that included both men and women in Ethiopia. i.e., Studies conducted on pregnancy-induced hypertension, and hypertension prevalence reports on other comorbidities

4). All articles were published in English, thesis paper, editorials, review, and conference papers were included in this review.

### Quality assessment and data collection

Joanna Brings Institute Meta-Analysis of Statistics Assessment and Review Instrument (JBI-MAStARI) was used for critical appraisal of the study. Newcastle-Ottawa scale was used for data extraction and quality assessment for this review.

Joanna retrieved studies were assessed for inclusion using their title and abstracts. Then a full review of articles for the quality of assessment was done before selecting for the final review. The details of studies that met the inclusion criteria were imported into the Joanna Briggs Institute's System for the Unified Management, Assessment and Review of Information (JBI SUMARI, The Joanna Briggs Institute, Adelaide, Australia) critical appraisal tools to evaluate the quality of all studies 18. All authors independently assessed the article title and abstract for inclusion in the review based on established article selection criteria, appraising the quality of the studies by criteria adapted for reporting prevalence data and cross-sectional studies. Studies were considered low risk if a score of 7 and above on the quality assessment indicators (Table 1.). Any discrepancy which arose between the reviewers were in the review process was solved through discussion with other reviewers.

**Table 1:** Critical appraisal results of eligible studies in the systematic review and meta-analysis on hypertension prevalence and associated factors in Ethiopia, 2021

S.no	Study	Q1	Q2	Q3	Q4	Q5	Q6	Q7	Q8	Q9	Total
1	Henok A., etal	Y	Y	N	Y	Y	Y	U	Y	Y	8
2	Akilew A., etal	Y	U	Y	U	Y	N	Y	Y	Y	7
3	Mihretie K., etal	Y	N	Y	Y	U	Y	y	Y	Y	8
4	Tsegab P., etal	Y	Y	Y	Y	Y	Y	Y	Y	Y	9
5	Zerihun A., etal	Y	U	Y	U	Y	Y	U	Y	Y	7
6	Solomon M., etal	Y	U	Y	Y	U	U	Y	Y	Y	7
7	Ufaysa A., etal	Y	U	Y	Y	U	Y	U	Y	y	7
8	Adefris C., etal	Y	Y	Y	Y	N	Y	Y	Y	Y	9
9	Belachew K., etal	Y	U	Y	Y	Y	Y	Y	Y	Y	8
10	Amare B., etal	Y	U	Y	Y	Y	U	Y	Y	Y	8
11	Daniel A., etal	Y	U	Y	U	Y	U	Y	Y	Y	7
12	Nebiyu D., etal	Y	Y	N	Y	Y	Y	U	Y	Y	8
13	Selamawit A., etal	Y	U	Y	U	Y	U	Y	Y	Y	7
14	Woldu A., etal	Y	N	Y	Y	U	Y	U	Y	Y	7
15	Gebrewahd B., etal	Y	Y	Y	U	Y	Y	Y	U	Y	8
16	Gemechis T., etal	Y	U	Y	Y	Y	Y	u	Y	Y	7
17	Feyie B., etal	Y	U	Y	Y	U	U	Y	Y	Y	9
18	Biniem H., etal	Y	Y	Y	Y	Y	Y	Y	Y	y	9
19	Demelash G., etal	Y	Y	Y	Y	N	Y	Y	Y	Y	9
20	Demelash W., etal	Y	U	Y	Y	Y	Y	Y	Y	Y	8
21	Solomon B., etal	Y	U	Y	Y	Y	U	Y	Y	Y	8
22	Yaregal A., etal	Y	U	Y	U	Y	U	Y	Y	Y	7

Y = Yes, N = No, U = Unclear; JBI Critical Appraisal Checklist for Studies Reporting Prevalence Data: Q1 = was the sample frame appropriate to address the target population? Q2. Were study participants sampled in an appropriate way? Q3. Was the sample size adequate? Q4. Were the study subjects and the setting described in detail? Q5. Was the data analysis conducted with sufficient coverage of the identified sample? Q6. Were the valid methods used for the identification of the condition? Q7. Was the condition measured in a standard, reliable way for all participants? Q8. Was there appropriate statistical analysis? Q9. Was the response rate adequate, and if not, was the low response rate managed appropriately?

### Data extraction

The data extraction was done using a tool developed by the 2014 Joanna Brings Institute Reviewers' Manual data extraction form by three authors (TL, BT, and MG) 18. The data extraction tool includes information on title, author, year of study, publication year, study design, sample size, study participants, study area, response rate, and the proportion of hypertension among adults in Ethiopia. Articles that fulfilled the predefined criteria were used as a source of data for the final analysis. The reviewers crosschecked it to ensure consistency. Any discrepancy was solved through discussion with other authors and the procedure was repeated to overcome the difference, which resulted during extracting every single study.

### Publication bias and heterogeneity

The existence of heterogeneity was assessed by using funnel plot test, I^2^ and its corresponding p-value. A value of 25%, 50%, and 75% was used to declare the heterogeneity test as low, medium, and high heterogeneity. For results with statistically significant heterogeneity, a random effect model of analysis was used. Egger regression asymmetry test was used to assess the statistical significance of publication bias 5.

### Data analysis

The data were entered using Microsoft Excel. The Meta-analysis was conducted using Stata 14 software. Forest plots were used to present the combined estimate with the 95% confidence interval (CI). The estimated pooled prevalence of hypertension among adults in Ethiopia was computed with 95% CI. Subgroup analysis was done by region, year of study period, and study participants. Additionally, the association of hypertension was conducted. Additionally, a univariate meta-regression model was applied by taking the sample size, publication year, and quality scores of each primary study to investigate the sources of heterogeneity. Finally, a forest plot figure was used to present the point proportions with their 95% CI of the primary studies.

## Results

### Studies identified

**Figure 1:**
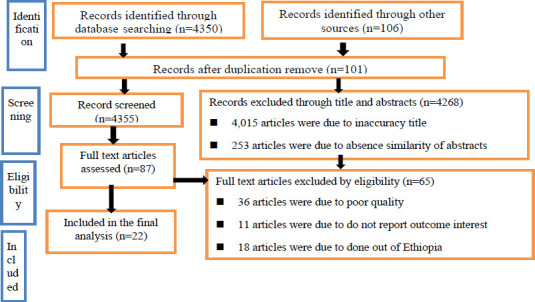
PRISMA flow diagram of selected and including studies for a systematic review and meta-analysis on the prevalence and associated factors of hypertension among adult population in Ethiopia, 2021

A total of 4350 articles were retrieved through internet searching. One hundred six identified through other sources. A total of 4456 articles were retrieved. Out of these, 101 duplicate records were removed from the review. From the total articles, 4,015 were due to inaccuracy title and 253 articles were due to absence similarity of abstracts were excluded from the review. After a full review of articles 65 were excluded by eligibility criteria. Finally, twenty-two studies were included in this meta-analysis (Fig.1.docx).

### Characteristics of included studies

This systematic review and meta-analysis included 22 articles with 14,202 study participants. All studies employed a cross-sectional study design. Most of the regions in Ethiopia were represented in this review. Two studies were from Addis Ababa city, seven studies from Amhara region, six from SNNRP, three from Oromia region, two from Tigray region, and one study from Afar and Harari region respectively. Studies were conducted from 2012 to 2021. The sample size of the studies ranged from 308 study conducted in Amhara region 4 to 3,346 a study conducted in SNNRP 19, and the response rate ranged from 93% to 100%. Overall, this review included 14,202 Adult hypertension clients in Ethiopia. (Table 2.).

**Table 2:** Characteristics of studies included in a Systematic Review on the Prevalence and associated factors of hypertension among adults in Ethiopia, 2021

Author	Year	Region	Study area	Study period	SD	Study group	Age (yrs.)	SS	Cases	PR (%)	RR (%)
Henok A., etal	2017	Afar	Jigjiga	Oct-Nov2014	CS	Residents	25–65	487	138	28.3	97.3
Akilew A., etal	2012	Amhara	Gonder	April 2012	CS	Residents	>35	679	192	28.3	97.6
Mihretie K., etal	2019	Amhara	Debre Markos	Feb-Mar, 2018.	CS	resident	>18	456	64	14	95.6
Tsegab P., etal	2014	SNNRP	Duram	April 2013	CS	Resident	>31	518	118	22.4	96.6
Zerihun A., etal	2018	Harar	Jugal Hospital,	Feb-April	CS	Patients	18- 68	425	54	12.7	95.9
Solomon M., etal	2015	Amhara	Dabat and Gondar	2012	CS	Resident	> 35	598	167	27.9	97.3
Ufaysa A., etal	2021	SNNRP	Areka town	Feb18-Mar 16, 2019	CS	Residents	≥25	576	110	19.1	99.2
Adefris C., etal	2020	SNNRP	Arba Minch	April to June 2017	CS	Residents	25-64	3,346	633	18.92	99.4
Belachew K., etal	2020	SNNRP	Arba Minch	Dec 1 to 30, 2017	CS	Residents	> 18	784	276	35.2	100
Amare B., etal	2018	Amhara	Felege-Hiwot	Mar 12-May 2, 2018	CS	Patients	>18	308	84	27.3	100
Daniel A., etal	2020	AA	Yekatit 12 Hospital	Sept-Oct. 2016	CS	Clients	>14	414	161	38.9	97.9
Nebiyu D., etal	2020	SNNRP	Hossana town	May15- 20, 2017	CS	Residents	≥18	627	108	17.2	98.9
Selamawit A., etal	2019	AA	Yekatit 12 Hospital	Nov-Dec 2015	CS	Clients	≥18	487	169	34.7	100
Woldu A., etal	2020	Tigray	Mekele Hospitals	Mar- May 2019	CS	Patients	≥18	391	190	48.6	98.7
Gebrewahd B.,etal	2019	Tigray	Ayder hospital	Feb 16–Apr 30,2018	CS	Patients	≥18	320	167	52.5	100
Gemechis T., etal	2019	Oromia	Nekemt	Nov 1 - Dec 30, 2015	CS	Residents	≥18	705	246	34.9	99.2
Feyie B., etal	2014	Oromia	Bedele town	July-Sept 2011	CS	Residents	>15	396	67	16.9	93.8
Biniem H., etal	2019	Amhara	Kombolcha	Dec2016–May 2017	CS	Residents	18-65	312	96	30.8%	100
Demelash G., etal	2021	Amhara	Debre Berhan	Feb 9, 2020-Mar 8, 2020	CS	Residents	18-65	680	187	27.5	100
Demelash W., etal	2020	SNNRP	Hawela	Feb to June 2019	CS	Residents	>18	383	47	12.3	98.3
Solomon B., etal	2021	Oromia	Jimma Town	June 17 - July27,2019	CS	Residents	>18	915	194	21.2	95.7
Yaregal A., etal	2018	Amhara	Gondar Hospital	Sep2015 - April 2016	CS	Clients	18-65	395	199	50.4	98

**NB:** AA=Addis Ababa, SD=study design, CS=cross sectional, PR=prevalence, RR=response rate

### The prevalence of hypertension among adults in Ethiopia

**Figure 2:**
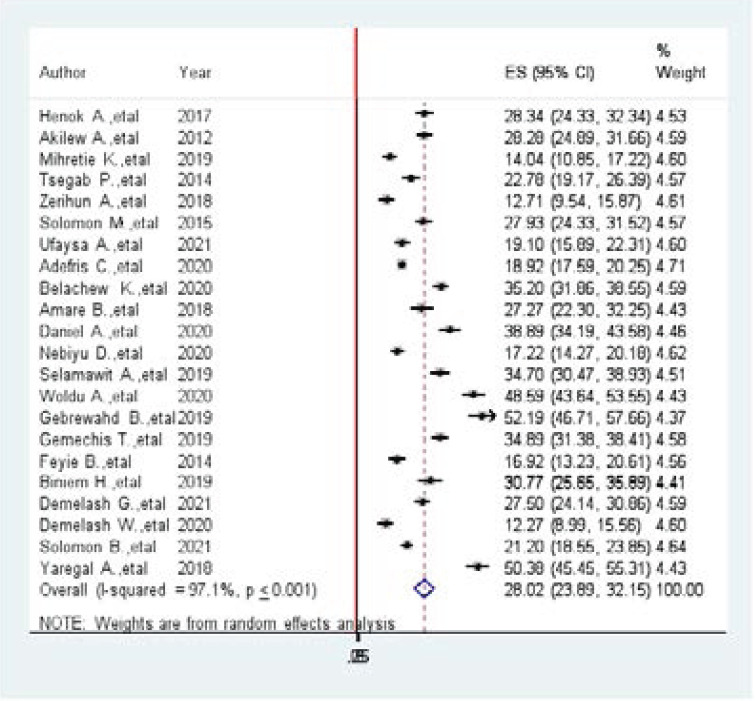
Forest plot of systematic review and meta-analysis on the prevalence of hypertension among adults in Ethiopia, 2021

Globally, the prevalence of hypertension among adults was observed in many studies included in this review. A prevalence of 12.3% in SNNRP 20 to 52.2% in Tigray region were observed 21. The I2 test result showed high heterogeneity (I^2^=97.1%, p-value < 001) which is indicative to use the random effects model of analysis. Thus, using the random effect analysis, the overall pooled prevalence of hypertension among Adults in Ethiopia was 23.76(23.08, 24.45) (Fig.2.docx).

### Subgroup analysis

**Figure 3:**
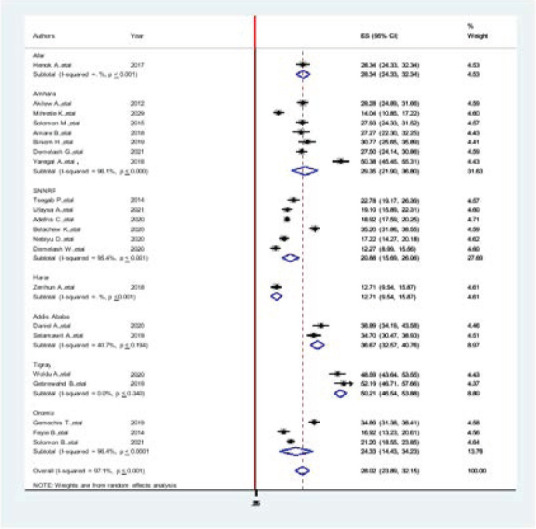
The pooled prevalence of hypertension in subgroup analysis among adult population in Ethiopia, 2021

Subgroup meta-analysis of the prevalence of hypertension among adults in Ethiopia was done by region showed that a higher pooled prevalence of hypertension among adults in Tigray region was 52.19(46.54, 57.66). The lowest prevalence of hypertension among adults was observed in Harari region12.71 (9.54, 15.87). The pooled prevalence of hypertension in subgroup analysis based on region was 28.02 (23.89, 32.15, I2= 97.1%, P < 0.001). The subgroup analysis done by publication year showed that a higher prevalence of hypertension was in a study done in 2019, 52.19(46.71, 57.66, I^2^=97.7%, P < 0.001). Lower prevalence of hypertension was in the year of 2020, 12.27(8.99, 15.56, I2=98.2%, p < 0.001) (Fig.3.docx).

### Factors associated with the prevalence of hypertension among adults in Ethiopia

A total of 22 primary studies had been shown as significant associated factor with the prevalence of hypertension among Adults in Ethiopia^4^,^6^,^26^–^35^,^7^,^36^,^19^–^25^. The identified associated factors in this reviewed study include age of the participants, sex of the study participants, family history of hypertension of the study participants, physical exercise of the study participants, body mass index (Obesity) of the study participants, khat chewing of the participants, salt and alcohol consumption of the study participants^4^,^6^,^26^–^35^,^7^,^36^,^37^,^19^–^25^.

### Association between age and hypertension among adults in Ethiopia

**Figure 4:**
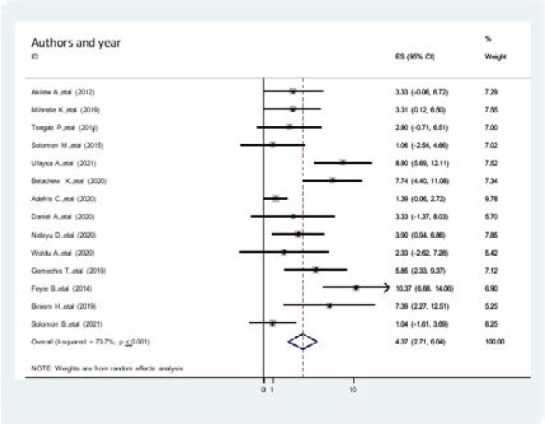
The association of age with the prevalence of hypertension among Adults in Ethiopia, 2021

The association between b/n age of the study participants and the prevalence of hypertension among Adults in Ethiopia was assessed by fourteen studies^6^,^19^,^29^–^35^,^20^,^22^–^28^. The meta-analysis of these fourteen studies demonstrated an increased odd of prevalence of hypertension with the age of the study participants among adults in Ethiopia. Overall, pooled prevalence of age associated with the prevalence of hypertension among Adults in Ethiopia was 4.37(2.71, 6.04, I^2^=73.7%, p < 0.000). The funnel plot is symmetrical, the observation association b/n, age, and prevalence of hypertension was not due to publication bias. The egger regression asymmetry test also demonstrated no statistically significant publication bias (Egger's test=0. 293, p < 0.030). (Fig.4.docx).

### Association between sex and hypertension among adults in Ethiopia

**Figure 5:**
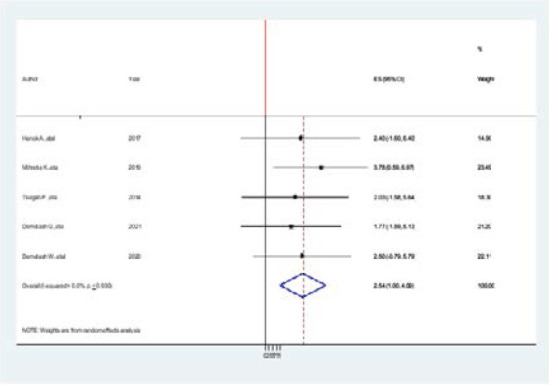
Forest plot of the association between sex and prevalence of hypertension among adults in Ethiopia, 2021

Five studies reported the association between the sex of the study participants and the prevalence of hypertension among Adults in Ethiopia^7^,^20^,^26^,^35^,^36^. The overall pooled AOR suggested that there was a significant association between sex and prevalence of hypertension (pooled AOR=2.54, 95% CI: 1.00–4.09, p < 0.930). This is 2 times greater than a study done in Member Countries of South Asian Association for Regional Cooperation(-sex (mean: odds ratio [OR] 1.19; 95% CI: 1.02, 1.37))^38^ (Fig.5.docx).

### Association between family history of hypertension and hypertension among Adults in Ethiopia

To identify the association between family history of hypertension and prevalence of hypertension among Adults in Ethiopia, ten studies were included in this meta-analysis 6,7,20,23,26,28,30,31,35,36.

**Figure 6:**
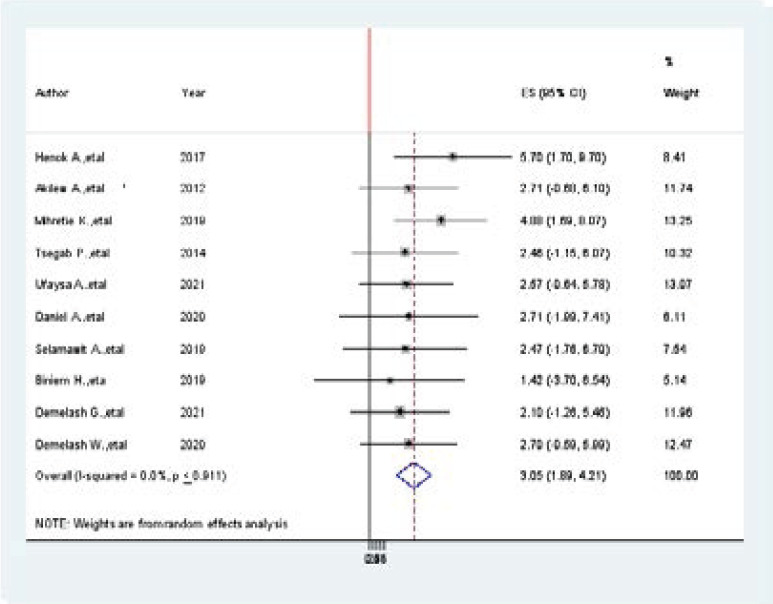
The pooled findings of the meta-analysis showed that having a family history of hypertension was significantly associated with the prevalence of hypertension among Adults in Ethiopia, 2021

Two of the included studies showed that having a family history of hypertension was significantly associated with the prevalence of hypertension and eight studies showed that there was no association between having a family history of hypertension and prevalence of hypertension among adults in Ethiopia. The pooled findings of the meta-analysis showed that having a family history of hypertension was significantly associated with the prevalence of hypertension among Adults in Ethiopia (AOR=3.05, 95% CI, 1.89, 4.21, I2=0.00, P < 0.911) (Fig.6.docx).

### Association between physical exercise and hypertension among adults in Ethiopia

**Figure 7:**
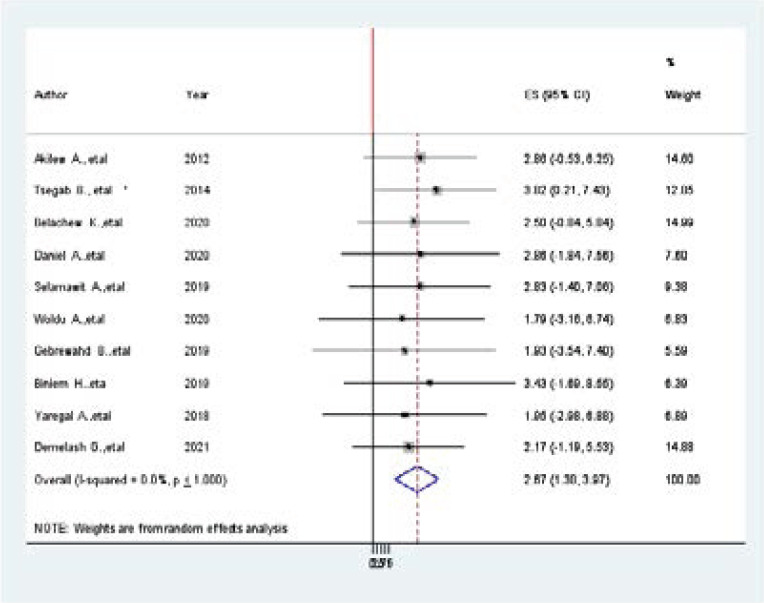
Association between physical exercise and prevalence of hypertension among Adults in Ethiopia, 2021

Ten studies were included in this meta-analysis and showed an association between physical exercise and prevalence of hypertension 6,7,32,34–36,19–21,23,26,28,30,31. Accordingly, from the included studies, none of them were showed a statistically significant association between prevalence of hypertension and physical exercise. The pooled findings of the meta-analysis showed that inactive physical exercise was significantly associated with the prevalence of hypertension among adults in Ethiopia (AOR=2.67, 95% CI: 1.38, 3.97). Since the funnel plot is symmetrical, the observation association between age and prevalence of hypertension was not due to publication bias. The egger regression asymmetry test also demonstrated no statistically significant publication bias (Egger's test=0. 478, p < 0.017) (Fig.7.docx).

### Association between obesity and hypertension among adults in Ethiopia

**Figure 8:**
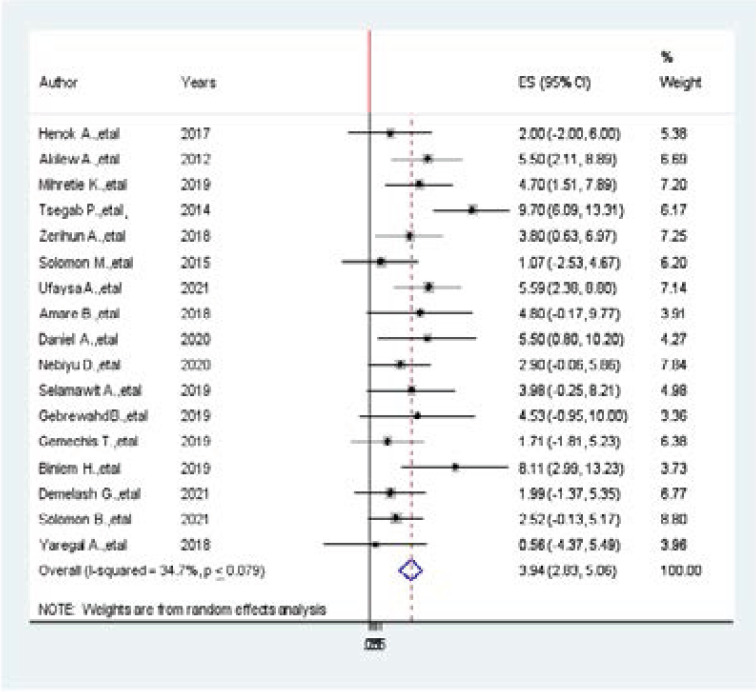
Forest plot of the association between obesity and hypertension among adults in Ethiopia

Seventeen primary studies were selected for meta-analysis and showed the association between b/n prevalence of hypertension among adults in Ethiopia and obesity of the study participants^2^,^6^,^30^-^36^,^7^,^12^,^19^–^21^,^23^,^26^,^28^. Five of them showed a positive association B/n obesity and prevalence of hypertension among adults in Ethiopia. The pooled findings of the meta-analysis showed that becoming more obese was significantly associated with the prevalence of hypertension (AOR=3.94, 95CI:2.83, 5.06, I^2^ =34.7%, p < 0.079) (Fig.8.docx). This is 2 times greater than a study done in in Member Countries of South Asian Association for Regional Cooperation (AOR 2.33; 95% CI: 1.87, 2.78)^38^. Funnel plot is symmetrical, the observation association B/n obesity and prevalence of hypertension was not due to publication bias. The egger regression asymmetry test also demonstrated no statistically significant publication bias (Egger's test=0. 330, p < 0.005).

### Association between khat chewing and hypertension among adults in Ethiopia

**Figure 9:**
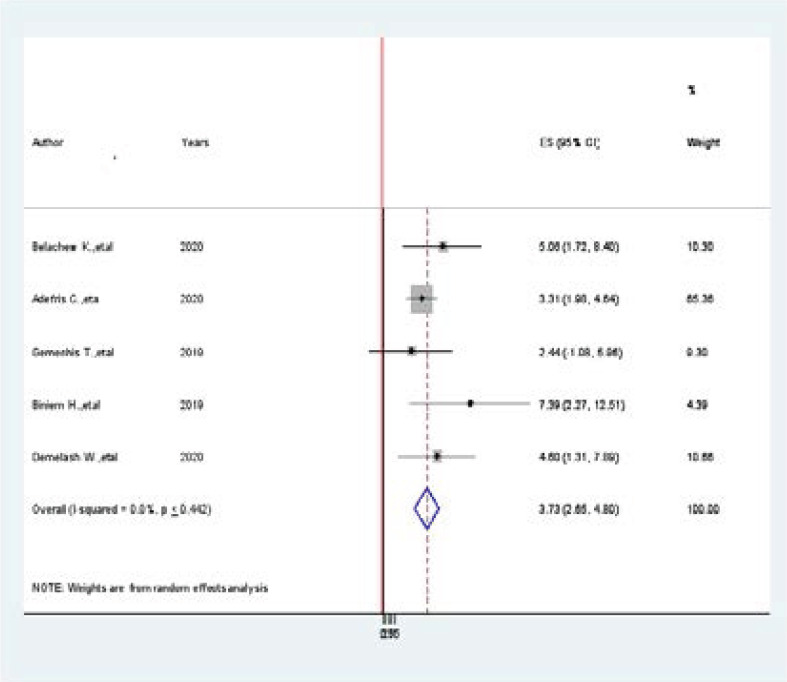
Forest plot of the Association between khat chewing and hypertension among Adults in Ethiopia, 2021

Five studies were included in this meta-analysis and showed an association between khat chewing and prevalence of hypertension 2,12,20,34,35. Accordingly, from the included studies, four of them were showed a statistically significant association b/n prevalence of hypertension and khat chewing. The pooled findings of the meta-analysis showing that khat chewing was significantly associated with the prevalence of hypertension among adults in Ethiopia (AOR=3.73, 95% CI: 2.65, 4.80, I^2^=0.00, p < 0.442) (Fig.9.docx). The egger regression asymmetry test also demonstrated no statistically significant publication bias (Egger's test=0. 0.256, p < 0.442).

### Association between salt consumption and hypertension among adults in Ethiopia

**Figure 10:**
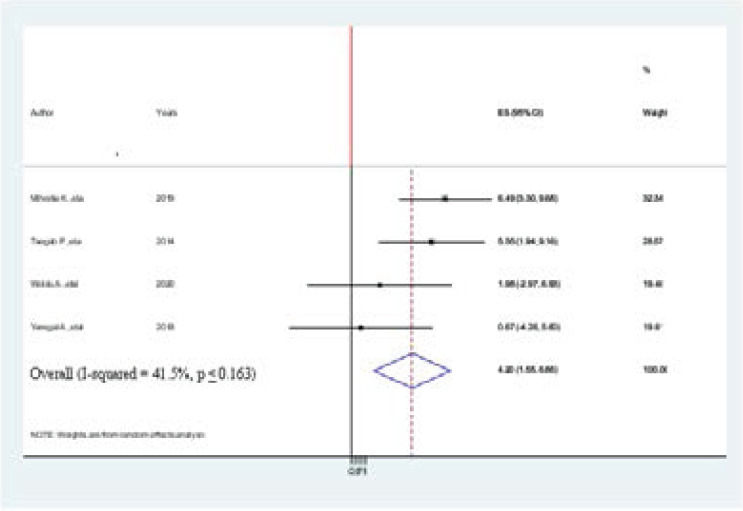
Forest plot of the association between salt consumption and hypertension among adults in Ethiopia, 2021

Four studies were included in this meta-analysis and showed an association between salt consumption and prevalence of hypertension^2^,^12^,^35^. Accordingly, from the included studies two of them were showed a statistically significant association between prevalence of hypertension and salt consumption. The pooled findings of the meta-analysis showing that salt consumption was significantly associated with the prevalence of hypertension among adults in Ethiopia (AOR=4.20, 95% CI: 1.55, 6.86) (Fig. 10.docx). The funnel plot is symmetrical, the observation association between salt consumption and prevalence of hypertension was not due to publication bias. The egger regression asymmetry test also demonstrated no statistically significant publication bias (Egger's test=1.358, p< 0.012

### Association between alcohol consumption and hypertension among Adults in Ethiopia

**Figure 11:**
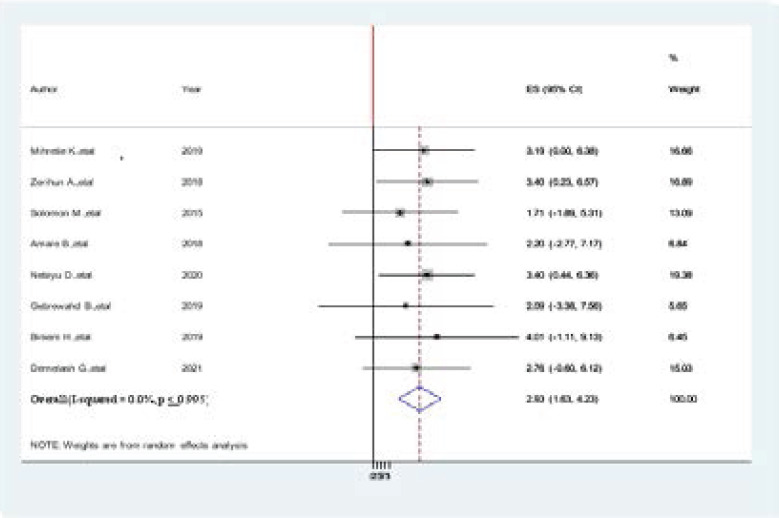
Forest plot of the association between alcohol consumption and hypertension among Adults in Ethiopia, 2021

Eight studies were included in this meta-analysis and showed an association between physical exercise and prevalence of hypertension^2^,^4^,^36^,^6^,^7^,^12^,^20^,^21^,^33^–^35^. Accordingly, from the included studies, none of them were showed a statistically significant association b/n prevalence of hypertension and physical exercise. The pooled findings of the meta-analysis showing that inactive physical exercise was significantly associated with the prevalence of hypertension among adults in Ethiopia (AOR=2.93, 95% CI: 1.63, 4.23) (Fig.11.docx).

## Discussion

This meta-analysis determined the pooled prevalence of hypertension among adult population in Ethiopia by addressing the major issue of hypertension prevalence and contributing factors that need to emphasize and get special attention from health care policy makers and health care professionals to early detect and management of this disease. After thorough searching and critically evaluating all these studies, we selected 22 studies with a total number of 14,202 study participants. Therefore, this systematic review and meta-analysis synthesized the findings of 22 studies that examined the prevalence and factors associated with hypertension among adults' population who are living in Ethiopia. The result of this review has the utmost significance to improve the quality of care for hypertensive patients by showing the summarized magnitude and suggesting possible strategies to improve the negative impact of hypertension.

The prevalence of hypertension among adults in Ethiopia was varied considerably across reports of primary studies that ranged from 12.3% to 52.5%. The overall pooled prevalence of hypertension among Adults in Ethiopia was 28.02% (95% CI: 23.89, 32.15). This finding was less than compared with previous systematic review and meta-analysis done on the prevalence of hypertension among adults in urban Asia population which stated that 32.45% 13. This variation might be due to the difference in study area in the primary studies included in reviews and the higher prevalence of hypertension in urban areas might be due to living lifestyle differences. Higher levels of obesity, increased salt and fat intake from consuming more processed foods, and engaging in jobs with minimal physical activity are possible explanations for higher hypertension in urban populations. The overall pooled prevalence of hypertension among Adults in Ethiopia was 28.02% (95% CI: 23.89, 32.15). Our review is lower than a study done in South Asia, specifically in SEA, a comprehensive review reported an adult hypertension prevalence of 35% 13. One plausible explanation is that these studies included different regions in Ethiopia only in comparison to these studies that includes different countries.

The overall pooled prevalence of hypertension among adult clients in Ethiopia was 28.02% (95% CI: 23.89, 32.15). This finding was higher than compared to a previous systematic review and meta-analysis done in 2015 with the prevalence of hypertension among the general population in Ethiopia was estimated to be 19.6 % 39 and to a previous systematic and meta-analysis done among hypertensive disorder of pregnant women in Ethiopia was estimated 6.07% 40. This variation might be due to study period, sample size, and study subjects.

The associated factors for hypertension can be categorized in two sociodemographic and modifiable risk factors. The sociodemographic-associated factors described in this review are sex, age, and family history of hypertension. In the present review, four articles mention that being in adult age is an associated factor of developing hypertension. In our review, five articles 2,4,12,20 mention that being male is a risk factor of developing hypertension. The overall pooled AOR suggested that there was a significant association between sex and prevalence of hypertension (pooled AOR=2.54, 95% CI: 1.00–4.09, p < 0.930). Similarly, a systematic review study done in member countries of South Asian association for Regional Cooperation is 2 times less than from our study (sex (mean: odds ratio [OR] 1.19; 95% CI: 1.02, 1.37)) 38. Accordingly, the possibility that there are fundamental causes elevated blood pressure and encourages sex convergence of hypertension prevalence caused by gender (e.g., psychosocial qualities such relative economic deprivation) or sex (e.g., sex hormones, chromosomal complement, pregnancy, or epigenetic changes). The mechanisms through which sex regulates the development of hypertension as well as its development itself are intricate and include many different systems. Recent developments in comprehension of the interaction between the oestrogen and testosterone and the rennin-angiotensin-aldosterone system (RAAS) shed light on the sexually dimorphic onset of hypertension 41.

This meta-analysis two articles ((12,20)) showed that having family history of hypertension was significantly associated with the prevalence of hypertension. The pooled findings of the meta-analysis showed that having a family history of hypertension was significantly associated with prevalence of hypertension among Adults in Ethiopia (AOR=3.05, 95%CI, 1.89, 4.21, I^2^=0.00, P < 0.911. However, a meta-analysis performed globally in middle and lower-income countries showed that there was no significant association between prevalence of hypertension and family history of hypertension among adult people living in Ethiopia.

On the other hand, modifiable associated factors including obesity, physical exercise, khat chewing, alcohol, and salt consumption are significantly associated with the prevalence of hypertension among adults in Ethiopia. In this meta-analysis, five articles showed that being obese is positively associated with the prevalence of hypertension among adults in Ethiopia. The pooled findings of the meta-analysis showing that becoming more obese was significantly associated with the prevalence of hypertension (AOR=3.94, 95CI:2.83, 5.06, I^2^ =34.7%, p < 0.079). Our review is 2 times greater strong associated than a study done in in Member Countries of South Asian Association for Regional Cooperation (AOR 2.33; 95% CI: 1.87, 2.78) 38. Accordingly, to this meta-analysis study, four articles showed a statistically significant association b/n prevalence of hypertension and khat chewing. The pooled finding of chat chewing was significantly associated with the prevalence of hypertension among adults in Ethiopia (AOR=3.73, 95% CI: 2.65, 4.80, I^2^=0.00, p < 0.442). Similarly, from the included studies, two studies showed a statistically significant association b/n prevalence of hypertension and salt consumption. The pooled finding of salt consumption was significantly associated with the prevalence of hypertension among adults in Ethiopia (AOR=4.20, 95% CI: 1.55, 6.86).

### Strengths of this review

The strength of this study includes the use of multiple databases to search articles (both manual and electronic search) for meta-analysis, abstraction of information uniformly in a predetermined format by two independent reviewers that helped to minimize error. This meta-analysis also included studies from different parts of the country that comprise both urban and rural populations.

### Limitations of this review

First, the bias may be present because the search was only done in the English language. Secondly, the categorization of certain variables, such as obesity, smoking, and level of physical activity, had different classifications across the primary reported studies, which made comparison of this review difficult. Moreover, all of the studies included in this review used a cross-sectional study design, which means that the outcome variable could be influenced by other confounding variables, lowering the study's power and making it more difficult to draw causal conclusions between associated factors and hypertension patient.

## Conclusions and recommendations

This review highlights the high prevalence of hypertension among adult people in Ethiopia. The study of hypertension is not only important because of its higher prevalence in the Ethiopian's region but also because of the fact that it is one of the most important modifiable risk factors for CVDs. This evidence suggests that Ethiopia should give attention and prioritization for early detecting and early managing of hypertension to reduce the future burden of the disease on the population. The rising prevalence of hypertension among the adult population must trigger policy makers and health care professionals to reduce chronic diseases like cardiovascular morbidity and mortality in the future. Further national population-based studies are required for a more accurate estimate of the prevalence of hypertension in the urban and rural population of the Ethiopia. In this meta-analysis, we reported that a number of modifiable risk factors such as obesity, khat chewing, and physical activity were associated with a high prevalence of hypertension in Ethiopia.

## Abbreviations

CVDs: Cardiovascular diseases
CDC: Centers for Disease Control and Prevention
WHO: World Health Organization
SNNPR: Southern Nation, Nationalities and Peoples of Region

## Data sharing statement

Data will be available by request to the corresponding author.

## Authors' Information

TL and BG are lecturers and practitioner nurses at the School of Nursing and midwifery, College of Medicine and Health Science, Wolaita Sodo University, Ethiopia. BT is lecturer at the department of Nursing, College of Medicine and Health Science, Wolkite University.
